# The clinical results of lower trapezius tendon transfer with the peroneus longus allograft augmentation combined with interpositional repair with fascia lata in massive irreparable rotator cuff tears

**DOI:** 10.15537/smj.2023.44.2.20220721

**Published:** 2023-02

**Authors:** Muhammet Bozoğlan, Murat Danışman, Tuğcan Demir, Halil Karaca, Cem Zeki Esenyel

**Affiliations:** *From the Orthopaedics and Traumatology Department (Bozoğlan), Izmir Health Sciences University Tepecik Training and Research Hospital, Izmir; and from the Orthopaedics and Traumatology Department (Danışman, Demir, Karaca, Esenyel), Prof. Dr. Ilhan Ozdemir Education and Research Hospital, Giresun, Turkey.*

**Keywords:** lower trapezius transfer, interpositional repair, massive tear, irreparable, rotator cuff tear, fascia lata

## Abstract

**Objectives:**

To investigate the clinical results of lower trapezius (LT) tendon transfer and interpositional repair that were performed simultaneously in patients with massive irreparable rotator cuff tears.

**Methods:**

Between 2018 and 2020 years, 16 patients with massive irreparable rotator cuff tears that were treated with LT tendon transfer and interpositional repair at the same time were included in this study. The mean follow-up period was 29±3 months (24-39 months) and the mean age of patients was 62±9 years (42-73 years). The acromio-humeral distance, active range of motions, Visual Analog Scale (VAS) scores, University of California-Los Angeles (UCLA) scores and Constant-Murley scores were made preoperatively and at the final follow-up.

**Results:**

At the final follow-up, forward flexion was increased from 109˚±24.7 to 144˚±22.21 (*p*=0.005), abduction from 60˚±16.33 to 135˚±16.33 (*p*=0.005) and external rotation from 12˚±16.87 to 35˚±14.34 (*p*=0.005). Total UCLA scores were 5.9±2.13 to 22.7±5.29 (*p*=0.005), Constant-Murley scores were 24±9.43 to 50.2±14.28 (*p*=0.008), VAS scores were 6.1±1.1 to 2.4±1.35 (*p*=0.007), mean acromio-humeral distances were 4.64±0.85 mm (3.42-6.23 mm) to 6.58 mm (5.25-8.21 mm) (*p*=0.005) preoperatively and at the final follow-up. Except one patient who had a frozen shoulder any significant complication was detected.

**Conclusion:**

Adding interpositional repair to the LT tendon transfer in patients with posterior superior irreparable rotator cuff tear seems to have satisfactory short to mid-term clinical outcomes without an increase in complications.

**R**otator cuff tears (RCT) are one of the shoulder injuries that cause pain, loss of function, and joint degeneration.^1^ The massive irreparable tear is defined as the presence of a tear in more than 2 tendons, retraction of the torn stump up to the glenoid level in the frontal plane (Patte stage 3), and advanced stage fatty degeneration (Goutellier stage 3-4).^2-4^

In patients with massive RCT who have waited for a long time, it is often impossible to repair the torn ends without creating tension. This tension on the tendon is one of the causes of unsuccessful repair and poor clinical outcomes.^5^ Good results have been reported with interpositional repair applications using grafts to reduce tension in the repaired tendon.^6^

Tendon transfers are among the treatment methods used in this patient group to restore shoulder functions and relieve clinical complaints. Lower trapezius (LT) tendon transfer was first defined and performed by Elhassan et al^7^ to restore shoulder functions in a patient with traumatic brachial plexus injury. Since the transfer is closer to the axis of motion of the posterior-superior rotator cuff muscles, the LT tendon can be harvested relatively easily, and the functional results are good, it has been increasingly preferred in the treatment of posterior-superior irreparable RCT.^8-12^

In LT tendon transfer, since there is an irreparable massive rotator cuff tear, the humeral head cannot be fully covered and a gap is formed. Repairing the retracted tendon with the interpositional method provides closure of the gap, and makes the procedure more anatomical.^13^

The hypothesis of this study was that in this patient group, which is difficult to treat, simultaneous application of LT tendon transfer and interpositional repair would both provide complete humeral head coverage and improve the clinical results.

## Methods

This retrospective, single-centre study was performed in the Department of Orthopaedic Surgery, Giresun University, Giresun Training and Research Hospital, Giresun, Turkey. Approval for the study was granted by the Clinical Research Ethics Committee of Giresun University Faculty of Medicine (Protocol Number: 2021/04, dated: 18.02.2021). All study procedures complied with the principles of the Helsinki Declaration. The study included 16 patients who underwent LT tendon transfer and simultaneous interpositional repair due to massive irreparable posterior-superior rotator cuff tear between January 2018 and January 2020 and had at least 2 years of follow-up. The inclusion criteria were: i) Retraction of the posterior superior cuff tear to at least the glenoid level on magnetic resonance imaging (MRI) (Patte stage 3 and advanced fatty degeneration (Goutallier stages 3 and 4); ii) Subscapularis tendon intact or the presence of a repairable tear; iii) Absence of glenohumeral arthrosis on radiographs (Hamada <3);^14^ and iv) No limitation in passive range of joint movements. The study exclusion criteria were defined as: i) the presence of additional comorbid diseases such as malignancy, a neurological deficit in the same extremity, uncontrolled diabetes, or chronic renal failure, ii) Low level of functional expectation, and iii) Patients who will not be able to adapt to the postoperative physical therapy process.

**Figure 1:**
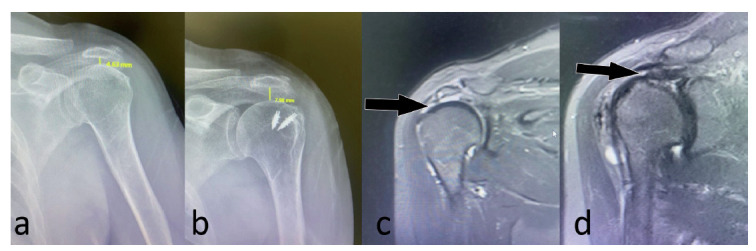
- Measurement method of acromio-humeral distance preoperatively (**a**) and postoperatively (**b**). Magnetic resonance views preoperative (**c**) and postoperative (**d**).

Preoperatively, direct radiographs (standard anteroposterior [AP] shoulder x-ray) and non-contrast MRI were taken of all patients. Preoperative and final follow-up acromio-humeral distance measurements were performed by the same person on standard AP shoulder radiographs (Figure 1 A & B). Passive shoulder range of motion in all directions was evaluated preoperatively. Active range of motion as forward flexion, abduction, and external rotation measurements were evaluated using a goniometer preoperatively and at the final follow up examination, together with the Visual Analog Scale (VAS), University of California Los Angeles (UCLA), and Constant-Murley scores.^15-17^

### Statistical analysis

All data were analyzed using the Statistical Package for the Social Sciences, version 21.0 software (IBM Corpn., Armonk, NY, USA). As the data of paired samples were non-parametric, the preoperative and postoperative scores were analyzed using the Wilcoxon signed-rank test. Relationships between parameters were evaluated with the Pearson correlation test. In all the comparisons, statistical significance was set at the level of *p*<0.05 (2-tailed).

### Surgical technique

**Figure 2:**
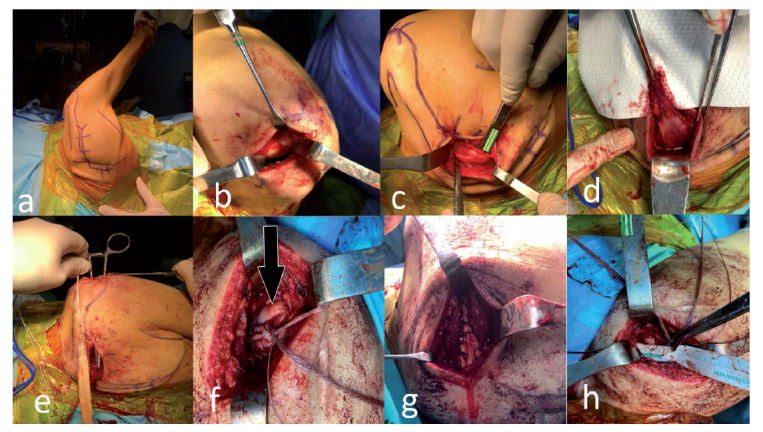
- Stages of the surgical procedure (**a**) Right shoulder of the patient with lateral decubitus position and landmarks. (**b**) The appearance of the subacromial space and humeral head after the acromioplasty. (**d**) Insertion area of the lower trapezius tendon to the scapula after the dissection of fascia. (**c**) Detaching lower trapezius tendon from the spine of scapula. (**e**) The path that was created between deltoid and infraspinatus (**f**) after fixing the allograft to the tuberculum majus the black arrow shows the gap on humeral head, (**g**) after suturing the allograft to the lower trapezius tendon, (**h**) measuring the gap on the humeral head.

All patients were operated under general anesthesia without interscalene block. The patients were placed in the lateral decubitus position with the affected side uppermost (Figure 2). The extremity to be operated on was prepared by sterilization with povidone iodine starting from the hand and up to the vertebral spinous processes and the sternum edge in the midline. At the same time, the ipsilateral thigh was sterilized from the proximal trochanter major region, including the 1/3 proximal part.

A 5 cm incision that the posterior edge was started in the center of the anteroposterior diameter of the acromion, and extended anteriorly toward the coracoid, just ending 1-2 cm beyond the anterior edge was used. The subcutaneous tissues were mobilized after the skin incision. After adequate skin mobilization, the anterior edge of the acromion was palpated. Using needle-flip electrocautery, the deltoid was detached from the anterior acromion subperiosteally, then the full-thickness flap of the deltoid was detached, including the superficial and deep fascia. The interval between the anterior and lateral deltoid was dissected longitudinally. The lateral and inferior acromioplasty was performed with an oscillating saw (Figure 2B).

**Figure 3:**
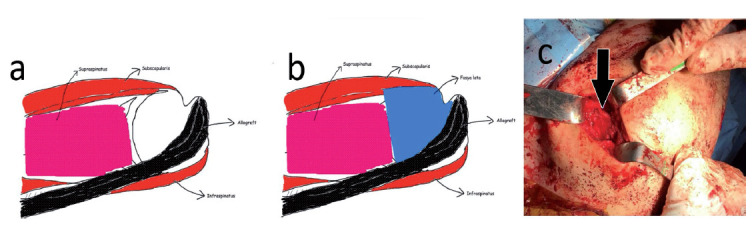
- Interpositional repair of the gap. (**a**) Shematic drawing of the gap on humeral head in patients with massive cuff tear after lower trapezius tendon transfer (**b**) and covering of the gap with fascia lata autograft. (**c**) The closed gap was shown with the arrow.

After the superficial and deep release of the rotator cuff muscles, tendon mobility was evaluated by using a grasper. If sufficient mobilization could not be achieved for the repair, a second incision was made starting 1 cm medial to the medial edge of the scapula extending to 3-4 cm lateral to the medial edge of the scapula for LT harvesting. Full-thickness skin flaps were created over the fascia of the LT (Figure 2C). The LT tendon was detached including the underlying periosteum from the spine of the scapula (Figure 2D). The LT muscle was mobilized from the middle trapezius. In this stage, care was taken not to injure the neurovascular pedicle that was located 2 cm medial to the scapula in the deep part of the muscle. A cleavage was created between the deltoid and infraspinatus muscles (Figure 2E). The next stage was the placement of the peroneus longus allograft, which had been previously sewn in Krakow style with number 2 stitches from both ends (peroneus longus 0.8 x 27.0 cm, milled dried, irrad, Maxxeus allograft). The allograft was first fixed to the border of the bicipital groove that was prepared before with a knotless anchor (Footprint Ultra PK Suture Anchor, 5.5 mm, Smith & Nephew). Then, 2-needle anchors (Twinfix, Ti 5.0 Suture Anchor with 2 38” Ultrabraid Sutures & Needles, Smith & Nephew) were placed on the prepared bed, one anteromedial and one anterolateral, after which the allograft was fixed using these anchors (Figure 2F). The allograft and LT muscle were fixed side to side when the arm was in maximum external rotation and abduction of 60° (Figure 2G). After the allograft was sutured to the infraspinatus muscle posteriorly, the gap that formed anterior to the allograft was measured (Figure 2H). A fascia lata autograft was taken from the ipsilateral thigh. It was prepared in 4 layers at the appropriate size and the measured gap was closed using the prepared graft. The graft was stitched anteriorly to the subscapularis muscle, medially to the retracted supraspinatus muscle, posteriorly to the allograft, and laterally to the anteromedial suture of the anchor, which was used to fix the graft (Figure 3 A-C).

All the patients were operated on by the same surgical team that had an experience of at least 10 years in shoulder surgery.

### Post-operative care

An arm sling was used by the patients for 6 weeks postoperatively with the shoulder in 30° abduction and 30° external rotation. The postoperative rehabilitation program consisted of 3 phases. The first phase (0-6 weeks) was the maximal protection stage, in which the main goals were minimizing pain, protecting the integrity of the repair and restoring pain-free passive range of motion (PROM). Active range of motion (AROM) exercises of the elbow, wrist, hand, and cervical spine were started immediately, and PROM of the shoulder in postoperative week 3. The second phase (6-12 weeks) was the AROM stage. The main goals of this stage were restoring functional AROM and proprioception, encouraging use of the operative upper extremity for light activities of daily living and successful weaning from the orthosis. Active assisted range of motion and AROM exercises were initiated in supine and side-lying positions, then progressed to antigravity positions as appropriate. The third phase was the strengthening stage with the main goals of regaining muscle strength and shoulder stability and enhancing optimal PROM/AROM.

## Results

The 16 patients comprised 12 (75%) males and 4 (25%) females with a mean age of 62.5±91 years (range, 42-73 years) and mean follow-up of 29±3 months (range, 24-39 months). Active forward flexion of patients was measured as 109°±24.7° (60°-140°) preoperatively and 144°±22.21° (90°-170°) postoperatively (*p*=0.005). The mean active abduction of the patients was 60°±16.3° (40°-90°) preoperatively, and 135°±16.3° (110°-170°) postoperatively (*p*=0.005). The mean degree of active external rotation was 12°±16.9° (-20°-30°) preoperatively, and 35°±14.3° (0-45) postoperatively (*p*=0.005) (Tables 1 & 2). The external rotation lag sign, which was positive in all the patients before the surgery, was evaluated as negative in all the patients in the postoperative period. Forward flexion was measured as 90° at the final follow-up examination in one patient with preoperative pseudoparalysis (anterior flexion 60°). During the procedures, the subscapularis tendon was observed as intact in all the patients.

**Table 1:** - Preoperative (preop) descriptive values of total UCLA scores, Constant Murley scores,VAS pain scores, acromiohumeral distance and active range of motions of shoulder (N=16).

Evaluated parameters	Minimum	Maximum	Mean	SD
Total UCLA score preop	3,00	10,00	5.90	2.13
Total Constant Murley score preop	15,00	45,00	24.00	9.43
VAS pain score preop	4,00	8,00	6.10	1.10
Acromiohumeral distance preop	3,42	6,23	4.64	0.85
Forward flexion preop	60,00	140,00	109.00	24.70
Abduction preop	40,00	90,00	60.00	16.33
External rotation preop	-20,00	30.00	12.00	16.87

UCLA: University of California Los Angeles VAS: Visual Analog Scale, SD: standard deviation,

**Table 2:** - Prostoperative (postop) descriptive values of total UCLA scores, Constant Murley scores,VAS pain scores, acromiohumeral distance and active range of motions of shoulder.

Evaluated parameters	Minimum	Maximum	Mean	SD
Total UCLA score postop	15,00	30,00	22.70	5.29
Total Constant_Murley score postop	19,00	68,00	50.20	14.28
VAS pain score postop	1,00	5,00	2.40	1.35
Acromiohumeral distance postop	5,25	8,21	6.58	0.84
Forward flexion postop	90,00	170,00	144.00	22.21
Abduction postop	110,00	170,00	135.00	16.33
External rotation postop	0,00	45,00	35.00	14.34

UCLA: University of California Los Angeles VAS: Visual Analog Scale, SD: standard deviation

The mean total UCLA score was 5.9±2.1 (3-10) preoperatively, and increased to 22.7±5.3 (15-30) postoperatively (*p*=0.005). The preoperative Constant-Murley score increased from 24±9.4 (15-45) preoperatively to 50.2±14.28 (19-68) postoperatively (*p*=0.008). The VAS pain scores were 6.1±1.1 (4-8) preoperatively, and 2.4±1.35 (1-5) postoperatively (*p*=0.007). The mean acromio-humeral distance was measured preoperatively as 4.64±0.84 mm (3.42-6.23 mm), and increased to 6.58 mm (5.25-8.21 mm) postoperatively (*p*=0.005) (Tables 1 & 2). It was observed that patients benefited from the surgery at a statistically significant level in all the parameters evaluated (Table 3).

**Table 3:** - Comparison of preoperative and postoperative values (Wilcoxon signed-rank test).

Evaluated parameters	Preoperative		Postoperative		Z	*P*
	Mean	SD	Mean	SD		
Total UCLA score	5.9	2.1	22.7	5.3	-2,81	0.005
Total Constant Murley score	24.0	9.4	50.2	14.3	-2,67	0.008
VAS pain score	6.1	1.1	2.4	1.4	-2,69	0.007
Acromiohumeral distance	4.6	0.9	6.6	0.8	-2,81	0.005
Forward flexion	109.0	24.7	144.0	22.2	-2,82	0.005
Abduction	60.0	16.3	135.0	16.3	-2,81	0.005
External rotation	12.0	16.9	35.0	14.3	-2,83	0.005

UCLA: University of California Los Angeles, SD: standard deviation

With the exception of one patient who had a cuff tear as a result of a physical attack and received psychological support, there were no observed complications. This patient was evaluated as adhesive capsulitis, was not able to comply with the postoperative rehabilitation process, and was subsequently lost to follow-up. Passive joint mobilization was performed under anesthesia in the 6th month postoperatively.

The preoperative and healed MRI views of one patient are shown in Figure 1 C & D.

## Discussion

The aim of this study was to report the clinical results of patients with massive RCT who were treated with LT tendon transfer and additional interpositional fascia lata autograft to close the gap on the humeral head. After considering the results which were reported previously in the literature, all the functional results in the present study group were determined to be sufficient and it was concluded that this procedure is quite useful.

El Hassan et al^18^ reported that the average shoulder flexion increased from 70˚ to 120˚, abduction increased from 40˚ to 90˚, external rotation increased from 20˚ to 50˚, and the patients had significant improvements in shoulder pain, subjective shoulder value (SSV), and disabilities of the arm, shoulder and hand (DASH) scores in the 47-month follow-up results of 33 patients who underwent LT tendon transfer with the open technique. In another 14-month follow-up series of 41 patients operated on arthroscopically, significant improvements were determined in shoulder movements, pain, subjective shoulder value (CSV), and DASH scores in 37 patients.^19^ Valenti and Werthel^20^ applied arthroscopy-assisted semitendinosus autograft to 14 patients in a series of LT tendon transfers, with a mean increase in active forward flexion of 150˚ to 160˚, external rotation from -20˚ to 24˚ with the arm at side, and external rotation from -10˚ to 40˚ when the arm was abducted at 90˚. The mean VAS and SSV scores increased significantly, and the external rotation lag sign, which was positive preoperatively, improved in all patients postoperatively.^20^ Stone et al^21^ reported that mean active forward flexion increased from 98˚ to 144˚, abduction increased from 74˚ to 127˚, and external rotation increased from 23˚ to 43˚ in 15 patients who underwent LT tendon transfer. Over a mean 2-year follow-up period, a significant improvement was reported in functional scores except for 3 patients who were revised with reverse shoulder prosthesis.

Shepherd et al^22^ reported that passive anterior flexion increased from 132˚ to 176˚, abduction increased from 131˚ to 176˚, and external rotation increased from 35˚ to 70˚, in the 9.7-year follow-up of patients who underwent interpositional repair with a synthetic patch graft. There was no significant difference between preoperative and outcomes in muscle strength evaluation. In a 50-month follow-up study of 61 patients who underwent interpositional repair with dermal xenograft, Neumann et al^23^ reported an increase from 140.7˚ to 160.4˚ in active forward flexion, from 55.6˚ to 70.1˚ in external rotation, and no significant difference in muscle strength values measured with a dynamometer compared to the healthy arm. The repair performed was reported to be intact in approximately 90% of the patients in the postoperative ultrasound evaluations.

In the present study, the average forward flexion increased from 109˚ to 144˚, abduction from 60˚ to 135˚, external rotation from 12˚ to 35˚, total UCLA score from 5.9 to 22.7, Constant-Murley score from 24 to 50.2, and the VAS pain score decreased to 2.1 from 6.4. With the exception of one patient, all the other patients were not able to put their forearms in external rotation position while the arm was by the side (the external rotation lag sign) preoperatively, although at the final follow-up examination, this deficiency was not observed in any patient.

Elhassan et al^18^ detected seroma formation in 4 patients after LT transfer with the open technique and reported that these patients improved with observation only. In another study of patients who underwent arthroscopic LT transfer, 3 seromas, one superficial infection, and 4 cases of hand hypoesthesia were observed but all patients recovered without any additional intervention.^19^ In the Valenti and Werthel^20^ series, 2 patients underwent revision due to hematoma in the grafted area (one had cutibacterium acne infection), but this complication was not seen to have an adverse effect on the results. Stone et al^21^ reported 2 patients who underwent debridement due to seroma and a patient who had cutibacterium infection and subsequently underwent reverse shoulder prosthesis. Neumann et al^13^ did not report any complications such as infection, inflammatory reaction, or tissue rejection in patients who applied dermal xenograft. In the present study, no complications such as seroma or infection were observed. In one patient who developed adhesive capsulitis due to lack of post-operative rehabilitation, a joint mobilization under anesthesia was necessary. Applying the 2 techniques together did not cause an increase in complications.

### Study limitations

This study is a retrospective design and has limited number of patients. Also, the follow-up period was short, and there was no control group treated with LT tendon transfer only.

In conclusion, the addition of interpositional repair with fascia lata autograft to LT tendon transfer in patients with posterior superior irreparable rotator cuff tear seems to be successful. Nevertheless, there is a need for further studies to compare the results of patients with and without interpositional repair.
